# Do the malnutrition universal screening tool (MUST) and Birmingham nutrition risk (BNR) score predict mortality in older hospitalised patients?

**DOI:** 10.1186/1471-2318-8-26

**Published:** 2008-10-10

**Authors:** Sarah Henderson, Nicola Moore, Emma Lee, Miles D Witham

**Affiliations:** 1 Department of Medicine for the Elderly, Royal Victoria Hospital, Dundee, UK; 2 Section of Ageing and Health, University of Dundee, Ninewells Hospital, Dundee, UK

## Abstract

**Background:**

Undernutrition is common in older hospitalised patients, and routine screening is advocated. It is unclear whether screening tools such as the Birmingham Nutrition Risk (BNR) score and the Malnutrition Universal Screening Tool (MUST) can successfully predict outcome in this patient group.

**Methods:**

Consecutive admissions to Medicine for the Elderly assessment wards in Dundee were assessed between mid-October 2003 and mid-January 2004. Body Mass Index (BMI), MUST and BNR scores were prospectively collected. Time to death was obtained from the Scottish Death Register and compared across strata of risk.

**Results:**

115 patients were analysed, mean age 82.1 years. 39/115 (34%) were male. 20 patients were identified as high risk by both methods of screening. A further 10 were categorised high risk only with the Birmingham classification and 12 only with MUST.

80/115 (67%) patients had died at the time of accessing death records. MUST category significantly predicted death (log rank test, p = 0.022). Neither BMI (log rank p = 0.37) or Birmingham nutrition score (log rank p = 0.35) predicted death.

**Conclusion:**

The MUST score, but not the BNR, is able to predict increased mortality in older hospitalised patients.

## Background

Undernutrition is known to be prevalent in hospitalized patients, with 40% of all patients admitted found to be undernourished[1]. Of those who are undernourished on admission, further deterioration often occurs during their inpatient stay[2]. Routine screening of nutritional state is therefore recommended in all patients admitted to hospital to allow early intervention.

Several different tools have been developed to screen for undernutrition. Two commonly used tools are the Birmingham Nutrition Risk Score (BNR) and the newer Malnutrition Universal Screening Tool (MUST). The former, the Birmingham Nutrition Risk Score was developed in the mid 1990's at the Birmingham Heartlands Hospital[3]. Initial validation studies showed that the score correlated well with other previously described scores, correlated well with a dietician's clinical impression and was reproducible between dieticians and nursing staff[4].

The more recent MUST score was developed by the Malnutrition Advisory Group of the British Association of Parenteral and Enteral Nutrition (BAPEN) for use in all health care settings. It also has excellent reproducibility between users, and is acceptable to patients and health care workers[5,6]. It too has been validated against a number of already accepted tools and was found to have good to excellent agreement with the BNR in the under 65 age group[7].

The MUST tool has now been adopted across most of the UK for screening nutritional risk. In September 2007 BAPEN undertook a Nutrition Screening Week to collect up to date data on nutritional risk across the UK. Overall malnutrition risk in hospitalised patients was 28%, with 22% of those being within the high risk category[8]. While the MUST tool identified a lower proportion than the anthropometric methods employed by McWhirter and Pennington[1], it still identified a significant number of undernourished patients. Age specific data for MUST showed that older hospitalised patients were more likely to be malnourished than younger people with 34% of people over 80 years malnourished[8]. 33% of patients on Medicine for the Elderly and stroke wards were at risk of malnutrition[8].

Despite the widespread acknowledgement that malnutrition causes adverse effects on physical and psychological function, little data is available on whether these screening tools identify older patients at high risk of adverse outcome[7]. Correct identification of these patients, who are at most need of nutritional intervention, allows appropriate targeting of scarce dietetic expertise and resources. Only one single centre study has examined the ability of MUST to predict mortality and length of stay in older people[9]. It is not clear from this study whether a high nutrition risk score was an independent predictor of either death or increased length of stay. The ability to predict mortality would suggest that a nutrition score has real prognostic value and is correctly identifying the group of patients at high risk of adverse outcome.

The aim of this study was therefore to test whether the MUST and BNR scores were able to predict mortality and length of stay in a cohort of older patients admitted to a specialist Medicine for the Elderly hospital.

## Methods

The study was carried out on the assessment wards at Royal Victoria Hospital, Dundee, UK. This is a specialist Medicine for the Elderly hospital, comprising 44 female and 30 male assessment beds. Referrals are accepted from the local community and from the nearby acute medical hospital. Patients with a wide variety of clinical presentations including falls, immobility, breathlessness, confusion and weight loss are admitted for assessment. Patients are accepted if aged 65 years or over, and likely to benefit from comprehensive geriatric assessment.

Prospective data were collected on all consecutive patients who were admitted to the male and female assessment wards between 10^th ^October 2003 to 9^th ^January 2004. No patients admitted during the study period were excluded. Nursing staff involved in assessing the patients on admission had all undergone training provided by their employer on assessing nutritional risk, since both scoring systems have been routinely used in our institution. Data relating to any previous amputation or the presence of oedema was corrected for, as described by the methods of the individual scoring systems. Patients were weighed by the member of nursing staff who admitted them, using Seca chair scales (Seca UK Medical Scales and Measuring Systems Ltd, Birmingham, UK). Each ward has its own set of scales, which are calibrated annually or sooner if there is any concern, by an external contractor. Patients were asked to recall their height but if unable to, were measured by the admitting nurse using a wall mounted measuring rod. In patients who were unable to stand, height was estimated using ulnar length measurements, as described in the MUST score methodology. Using these data, Body Mass Index (BMI), MUST and BNR scores were calculated by the admitting nurse, as part of the normal admissions procedure. Although weight and BMI are components of both scores, it was possible in some cases to assign patients to high risk categories based on the cumulative score of the remaining components. On admission, patients were asked to self report any unplanned weight loss. In those patients in whom it was unclear, old case notes were obtained and any previous weights sought to clarify this. Each patient was then categorised as being of low, medium or high risk of undernutrition using each one of the scores studied. Since the data were collected as part of the admission procedure, nursing staff were blinded to the eventual outcome.

On 1^st ^July 2006, data were obtained from the Scottish Death Register on date and cause of death for these patients if applicable. The Scottish Death Register holds details of date and cause of death as recorded by clinicians on the death certificate. Length of stay data was retrieved from the departmental admissions database for all patients. All data were recorded on a Microsoft Excel database and transferred to SPSS v15 for analysis. A follow-up period in excess of two years was chosen to ensure that enough deaths would have occurred for meaningful statistical analysis. Kaplan-Meier Plots were used to compare mortality in the low, medium and high risk groups as scored by the individual screening tools. These strata were then compared using log rank tests. Cox regression analysis was performed adjusting for age and sex to calculate the mortality hazard ratios for each category, with low risk being the referent category for each tool. Spearman's rho test was used to test the correlation between length of stay and risk category for each nutrition risk tool.

These data formed part of an ongoing internal audit within our department using routinely collected clinical data, and therefore in the opinion of Tayside Research Ethics Committee, did not require formal ethical approval [see Additional file 1].

## Results

126 consecutive patients were admitted during the study period of 10^th ^October 2003 to 9^th ^January 2004. Data needed to calculate Body Mass Index (BMI) were missing in 11/126 patients (8.7%). For the remaining 115 patients, the mean age was 82.1 years (SD 7.0). 39/115 (34%) were male, and the median MSQ was 8 (IQR 4.25). 78/115 (68%) were admitted from home, 13/115 (11%) were admitted from sheltered housing, 6/115 (5%) from residential homes, 4/115 (3%) from nursing homes, 2/115 (2%) from long stay medical wards and 12/115 (10%) were transferred from an acute hospital.

Information was collected from the routine nursing admissions procedure and therefore some data were missing. 114/115 (99%) patients had a BNR score recorded. Based on this score, they were categorised into low, medium and high risk as shown in Table 1. BMI was available in all 115 patients and was used to categorise patients into World Health Organisation categories[10], as shown in Table 1. 92/115 (80%) patients also had a MUST score recorded, and similarly, were categorised into low, medium and high risk based on this scoring system, as shown in Table 1. The commonest reason for not being able to calculate a MUST score (n = 13) was a lack of information on weight loss or any record of previous weights.

**Table 1:** Number of Patients in each category of Nutrition Risk Scores.

*BMI*	*Number of Patients (%)*
BMI <18.5 (underweight)	17/115 (15%)
BMI 18.5 – 24.9 (normal weight)	62/115 (54%)
BMI 25 – 29.9 (overweight)	26/115 (23%)
BMI 30–39.9 (obese)	9/115 (8%)
BMI >40 (morbid obesity)	1/115 (1%)

*Birmingham Nutrition Risk Score*	

Low Risk = 0–3	45/114 (39%)
Medium Risk = 4–5	33/114 (29%)
High Risk = 6–15	36/114 (32%)

*MUST Score*	

Low Risk = 0	47/92 (51%)
Medium Risk = 1	13/92 (14%)
High Risk = 2 or more	32/92 (35%)

Of the patients with both MUST scores and NRS scores, 42/92 (46%) were categorised as high risk by one or both scores. 10/42 (24%) were at high risk on the BNR score but not the MUST score, 12/42 (29%) were at high risk on the MUST score but not the BNR score, and 20/42 (48%) were at high risk on both scores.

**Figure 1:**
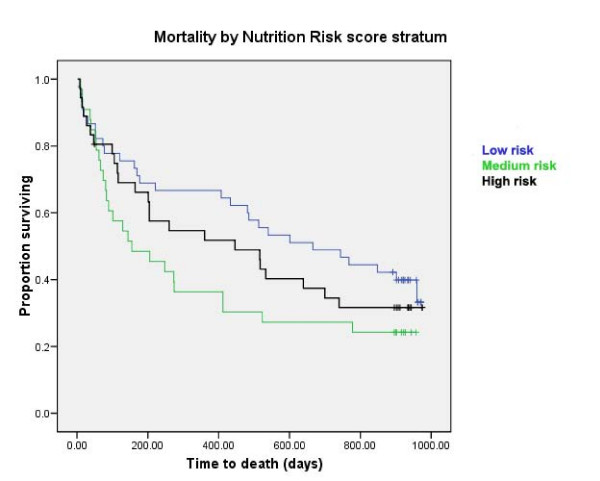
**Survival curves stratified by risk category of BNR score**. Log rank: Chi-square 0.86, df = 1, p = 0.35.

**Figure 2:**
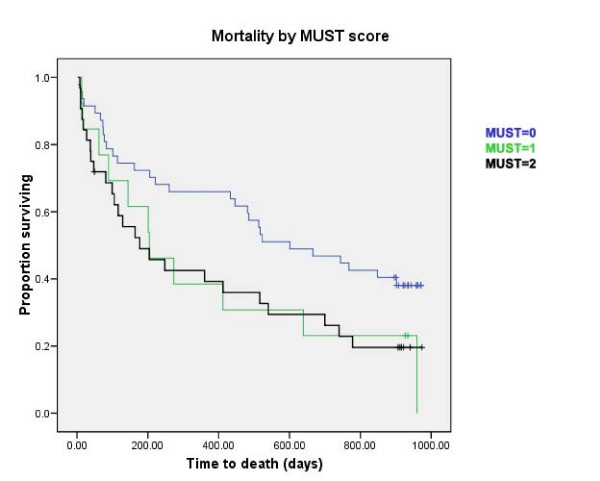
**Survival curves stratified by risk category of MUST score**. Log rank: chi-squared 5.28, df = 1, p = 0.022.

77/115 (67%) patients had died at the time of accessing death records; data are given in Table 2. Estimated median time to death from admission was 446 days (95% confidence interval 234 to 658 days). Median length of stay was 23 days (interquartile range 32 days; range 0–433 days). Survival curves stratified by risk category are shown in Figs 1 and 2. MUST risk category was shown to significantly predict mortality (log rank test, *p *= 0.022), whereas the BNR did not (log rank, *p *= 0.35). Furthermore, no statistically significant association was identified between BMI at admission and mortality (log rank *p *= 0.37). Table 2 gives hazard ratios for mortality calculated using Cox regression analysis adjusted for age and sex. Length of stay correlated with BMI category (r = -0.27, *p *= 0.003, Spearman's rho), but not with either MUST (r = 0.10, *p *= 0.37, Spearman's rho) or BNR (r = 0.10, *p *= 0.27, Spearman's rho) risk categories.

**Table 2:** Adjusted hazard ratios for mortality by malnutrition category

*BMI*	*Number dead at follow up (%)*	*Adjusted hazard ratio (95% CI)*
BMI <18.5 (underweight)	11/17 (65%)	1.05 (0.54 to 2.04)
BMI 18.5 – 24.9 (normal weight)	44/62 (71%)	1.00
BMI 25 – 29.9 (overweight)	16/26 (62%)	0.67 (0.38 to 1.19)
BMI 30–39.9 (obese)	5/9 (56%)	0.89 (0.33 to 2.35)
BMI >40 (morbid obesity)	1/1 (100%)	0.85 (0.11 to 6.55)

*Birmingham Nutrition Risk Score*		

Low Risk = 0–3	28/45 (62%)	1.00
Medium Risk = 4–5	25/33 (76%)	1.74 (1.01 to 3.01)
High Risk = 6–15	24/36 (67%)	1.17 (0.68 to 2.05)

*MUST Score*		

Low Risk = 0	29/47 (62%)	1.00
Medium Risk = 1	11/13 (85%)	1.91 (0.95 to 3.83)
High Risk = 2 or more	25/32 (78%)	1.98 (1.15 to 3.42)

## Discussion

This is the first study to compare MUST with the BNR in the over 65 age group. By using mortality as an endpoint in this study we have shown that MUST is not simply an abstract tool but can be related to a real clinical outcome and thus has prognostic value when screening for nutritional risk in older patients. If patients defined as high risk in the MUST screening tool have a higher incidence of death, this suggests that targeting nutritional interventions at those at high risk would be more likely to be able to reduce mortality. Furthermore, our data suggest that patients with an intermediate risk on the MUST score have similar increased mortality as those in the high risk group.

Although there was some overlap between patients categorised as high risk by the MUST and BNR tools, some patients were categorised as high risk on only one tool. Our data suggest that the MUST, but not the BNR, is correctly identifying patients that are at highest risk of death. One possible reason for the lack of congruence between the tools is the fact that MUST contains specific questions about acute illness, whereas the BNR contains questions skewed towards detecting chronic illness[3].

Little validation work has been performed on the BNR score; the original study[3] describing the tool validated it by comparison with another nutrition assessment tool, but data on the ability of the BNR to predict outcomes in older people are lacking. In one study comparing the BNR to anthropometric measurements of undernutrition, the BNR failed to detect a fifth of cases of undernutrition[11], suggesting that misclassification may reduce the predictive power of the BNR tool. Considerably more work has been done to validate the MUST tool; it has been shown to have good inter-rater agreement, and good agreement with a full dietetic assessment for detecting malnutrition[6]. The MUST score has also been found to correlate well with a series of other tools including the mini nutritional assessment and the BNR[7]. In contrast to our findings, the BNR and MUST scores showed good correlation with each other when compared in younger medical inpatients; this difference in population age, plus the fact that medical undergraduates rather than nursing staff administered the tools, may account for the difference. One other study has examined the ability of the MUST score to predict death in hospitalised older adults[9]; this also showed that patients with high MUST scores had higher death rates. Interestingly, all patients enrolled in this study could have a MUST score completed; in the absence of weight data, subjective recall of weight loss was used with greater success than in our population group. It is possible that better data collection in the context of a research study, plus screening out those with severe cognitive impairment who would be unable to give consent may account for these differences.

Despite all of the nursing staff in our institution receiving standardised training on nutritional risk screening some patients did not or could not, owing to their medical condition, have this data recorded. This is however, a reflection of real life practice in hospitals and is a limit to any scoring system. Reliable information on recent weight loss was not obtainable in some patients, which precluded calculation of an accurate MUST score.

Patients in this study were categorised into risk strata depending on the methodology of each individual tool. It is indeed possible that patients could have been misclassified[11], but we did not collect anthropometric data, the gold standard of measurement, with which to validate the categorization; this was not the aim of the present study. Whilst there is some data that corrected arm muscle area has better prognostic value than BMI alone with lower corrected arm muscle area giving an increased risk of mortality at 8 years[12]. This technique however involves the collection of anthropometric data that is unlikely to be suitable for use in routine clinical practice by nursing staff.

Whilst nutritional status is clearly not the only risk factor for death in older frail patients, the failure of the BNR score to correlate with mortality suggests that this tools lacks predictive validity for use in this patient population[13]. Worse still, patients at high risk on the BNR had lower mortality than those at medium risk. Our analysis showed that there was a statistically significant difference in mortality between the MUST high and low risk scores, while there was no statistically significant difference between any of the categories of the BNR score.

We are unable to adjust further for comorbid disease as we did not collect detailed information on comorbidity or physical and psychological indices of frailty. Whilst a relatively long follow up time was employed to ensure adequate numbers of deaths to study, the ability of nutritional tools to predict short-term mortality would arguably be even more useful, especially in directing early nutritional intervention to those most in need of it. Larger studies in the future would give more power to the detection of short-term mortality; such studies could also collect further data on co-morbid disease and measures of frailty that would allow for adjustment of these possible confounding factors. Care is needed to ensure that adjustment for frailty and comorbidity does not in fact obscure a true underlying association between malnutrition and mortality however.

## Conclusion

Our results lend support to the current drive to use the MUST tool as a screening tool in all healthcare settings in Scotland and beyond, and give confidence that the tool is detecting a group of patients at high risk of death.

## Competing interests

The authors declare that they have no competing interests.

## Authors' contributions

MW, NM and EL planned the study and carried out data collection. SH followed up patients and analysed the data. MW and SH cowrote the paper; NM and EL critically revised the manuscript. All authors read and approved the final manuscript.

## Pre-publication history

The pre-publication history for this paper can be accessed here:



## Supplementary Material

Additional file 1 Click here for file
