# Supplementary material for: Do the malnutrition universal screening tool (MUST) and Birmingham nutrition risk (BNR) score predict mortality in older hospitalised patients?
Source: BMC Geriatr. 2008 Oct 10;8:26. doi: 10.1186/1471-2318-8-26 (PMC2572058; doi:10.1186/1471-2318-8-26)
Supplement: Additional file 1 [file 1471-2318-8-26-S1.pdf]

Tayside Committee on Medical Research Ethics A
Level 9
Ninewells Hospital & Medical School
DUNDEE
DD1 9SY
Telephone Number: 01382 632701
Fax Number: 01382 496207
www.nhstayside.scot.nhs.uk

NHS Tayside

Dr Miles D Witham
Clinical Lecturer
Section of Ageing & Health
Department of Medicine & Therapeutics
Ninewells Hospital & Medical School
DUNDEE
DD1 9SY

Date	27 February 2006
Your Ref	
Our Ref	NFB/FB/
Enquiries to	Mr Nigel F Brown
Extension	32701
Direct Line	01382 632701
Email	nigel.brown@tuht.scot.nhs.uk or fiona.bain@tuht.scot.nhs.uk

Dear Dr Witham

Re: Do the MUST and Birmingham Nutrition Risk Scores predict mortality in older hospitalised patients?

Thank you for your letter of 15 February 2006 with which you enclosed a copy of the protocol in connection with the above.

I have discussed this with the Medical Advisor and we are of the opinion that this does not raise any ethical issues and therefore does not require to be submitted to the Research Ethics Committee. However, we would advise you that you should obtain Caldicott Guardian approval for access to the patients' case notes.

Yours sincerely

Miss Fiona Bain
Assistant REC Administrator
